# Comparison of Effectiveness of Betamethasone gel Applied to the Tracheal Tube and IV Dexamethasone on Postoperative Sore Throat: A Randomized Controlled Trial

**Published:** 2013-10

**Authors:** Masoomeh Tabari, Ghasem Soltani, Nahid Zirak, Mohammad Alipour, Kamran Khazaeni

**Affiliations:** 1 *Department of Anesthesiology, Mashhad University of Medical Sciences, Mashhad, Iran.*; 2 *Oral and Maxillofacial Diseases Research Center, Mashhad University of Medical Sciences, Mashhad, Iran.*

**Keywords:** Betamethasone, Dexamethasone, Pharyngitis, Surgical procedures complications, Tracheal intubation

## Abstract

**Introduction:**

Postoperative sore throat is a common complaint in patients with endotracheal intubation and has potentially dangerous complications. This randomized controlled trial study investigated the incidence of postoperative sore throat after general anesthesia when betamethasone gel is applied to a tracheal tube compared with when IV dexamethasone is prescribed.

**Materials and Methods:**

Two hundred and twenty five American Society of Anesthesiologist (ASA)-class I and II patients undergoing elective abdominal surgery with tracheal intubation were randomly divided into three groups: betamethasone gel, intravenous (IV) dexamethasone, and control groups. In the post-anesthesia care unit, a blinded anesthesiologist interviewed all patients regarding postoperative sore throat at 1,6, and 24 hours after surgery.

**Results:**

The incidence of sore throat was significantly lower in the betamethasone gel group compared with the IV dexamethasone and control groups, 1, 6, and 24 hours after surgery. In the first day after surgery 10.7% of the betamethasone group had sore throat whereas 26.7% of the IV dexamethasone group and 30.7% of the control group had sore throat. Bucking before extubation was observed in 14(18.4%), 8(10.4%), and 9(12.2%) patients, in the IV dexamethasone, betamethasone gel, and control group, respectively.

**Conclusion:**

We concluded that wide spread application of betamethasone gel over tracheal tubes effectively mitigates postoperative sore throat, compared with IV dexamethasone application.

## Introduction

Postoperative sore throat is a common complaint and an uncomfortable side effect in patients receiving general anesthesia with endotracheal intubation, with a reported incidence of 15–90% (1–4). Postoperative sore throat could be a symptom of potentially dangerous complications such as hypertension, cardiac dysrhythmia, myocardial ischemia, surgical bleeding, bronchospasm, increased intraocular, or intracranial pressure (5,6). 

The following factors result in a sore throat due to irritation and inflammation of the airway (4); trauma to the pharyngolaryngeal mucosa (7), cuff design, contact of the tracheal tube with vocal cords, cuff form, pressure-induced tracheal mucosal capillary hypo perfusion (8), and pressure over the posterior pharyngeal wall resulting in edema and mucosal lesions (9).

Several interventions prevent postoperative sore throat, such as use of endotracheal tubes of smaller sizes (9), lower intra-cuff pressure (10,11), use of steroid-coated tubes (13), application of topical lidocaine (12), and inhalation of steroids postoperatively (14). 

Recently, postoperative administration of the potent corticosteroid IV dexamethasone has been confirmed to offer analgesic and anti-inflammatory effects and an antiemetic action (15). This intervention reduced the incidence and severity of postoperative sore throat in patients receiving general anesthesia with endotracheal intubation (16). Its prophylactic use significantly decreased the incidence and severity of sore throat after tracheal extubation (17). 

The anti-inflammatory role of steroids has been demonstrated in previous studies (3,9). The application of betamethasone gel, therefore, to the tracheal tube might reduce the incidence of postoperative sore throat, cough, and hoarseness (4,18). It has been proved that applying local anesthetic jelly due limits potential damage to the tracheal mucosa because its lubricating properties suppress bucking on the tracheal tube (19). This study aimed to evaluate the incidence of postoperative sore throat when betamethasone gel is applied to the tracheal tube. Previous small studies compared betamethasone gel and placebo groups (4,18), or betamethasone gel and lidocaine (20). Therefore, we decided to compare the incidence of sore throat following application of betamethasone gel to the tracheal tube against IV dexamethasone and standard saline as the control groups in a randomized controlled trial study. 

## Materials and Methods

The study was initiated following approval from the Institutional Ethics Committee of Mashhad University of Medical Sciences. Signed informed consent was provided by all patients. A total of 225 patients with American Society of Anesthesiologist (ASA) values varying between I and II were enrolled. All patients were aged between 20–45 years and were scheduled for elective abdominal surgery with tracheal intubation under general anesthesia. 

Patients with a positive history of cardio-respiratory or cerebro-vascular disease; a background of chronic allergic diseases; pulmonary diseases such as asthma; gastro-esophageal reflux; renal or hepatic failure; hyper- or hypothyroidism; hypertension; preoperative sore throat; preoperative analgesic and anti-inflammatory drugs; substance addiction; beta-blocker users; and patients under 20 years old were all excluded. In addition, more than two attempts at intubation, use of nasogastric tube or throat packs during surgery, upper respiratory tract infection, steroid therapy, and pregnancy were exclusion criteria. Pre-anesthesia evaluation was performed and then selected participants were divided into three groups with 75 patients in each group using a standard randomization method.

- Group one: Betamethasone group (i.e. 0.05% betamethasone gel applied over the tracheal tube).
- Group two: IV dexamethasone
- Group three: Control group (i.e.standard saline applied over the tracheal tube).

All subjects were pre-medicated with IV midazolame (40 µ/kg/ before intubation), and routine monitoring was used for all patients, including non-invasive blood pressure (NIBP), pulse oximetry, electrocardiography (ECG) and capnography. Induction of anesthesia was performed by fentanyl (3-4 µg/kg) Propofol (2mg/kg) was administered over 30 s followed by IV atracarium (0.5mg/kg). Intubation was performed by a single expert anesthesiologist using a Macintosh laryngoscope. An appropriate laryngoscope blade was selected for each patient according to the patient’s body size (tracheal tube size of 7 mm for women and 8 mm for men). In both groups, anesthesia was maintained by 50% O2 and 50% nitrous oxide, propofol (100 µg/kg/min) and fentanyl (0.0–-0.04 µ/kg/min) infusion. The trachea was extubated after deflating the cuff when the patient was fully awake. All patients received oxygen through a facemask after the operation and underwent a similar hydration regimen (serum ringer initiated in all patients before induction with 5 ml/kg before it was continued during the operation based on individual needs).

Assessment of postoperative sore throat was carried out at 1, 6, and 24 hours after surgery by a blinded anesthesiologist using a questionnaire

***Statistical analysis***

Statistical analysis was performed using SPSS software, version 11.5 (SPSS Inc., Chicago, IL, USA). Patients’ characteristics such as heart rate (HR), systolic blood pressure (SBP), diastolic blood pressure (DBP) and MAP (Mean Arterial Pressure) were analyzed by analysis of variance (ANOVA). (Fisher’s F-test) or Kruskal Wallis t-tests were used to compare variables between each group wherever appropriate. 

Numerical data are expressed as mean ± standard deviation (SD) or as proportions of the sample size. Categorical data were analyzed by χ2 test. Statistical significance was considered as P<0.05. Considering the results of a previous study that showed an incidence of 50% for sore throat after lubrication with placebo gel, we calculated that 73 patients would be required in each group to detect a difference of 20% in the incidence, with power of 80% and a=0.05 (20). 

## Results

During the study, no patient was excluded from analysis. Patients’ characteristics are shown in Table 1. There was no significant statistical difference between the groups with respect to age or duration of surgery (P>0.05). There was no significant difference between the studied groups in measured hemodynamic indices (SBP, DBP, or HR; P>0.05). 

**Table 1:** Patients characteristics, data are mean (SD).

	**Variable**	**Betamethasone (n=75)**	**Dexamethasone (n=75)**	**Control ** **(n=75)**	**P-value**
**Group**					
Age		42.35 (11.15)	41.5 (14.26)	40.52 (11.57)	(P>0.05)
Sex (female/male)		68 (7)	58 (17)	63 (12)	(P>0.05)
Operation duration (min)		105(11)	100(9)	98(10)	(P>0.05)

**Fig 1:**
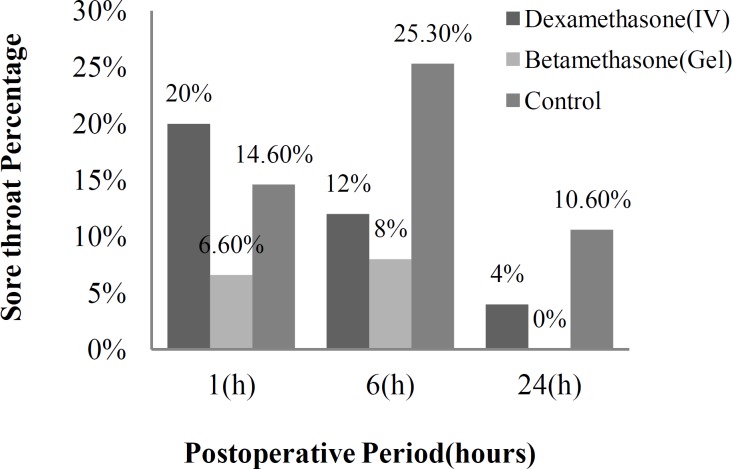
Incidence of sore throat. Data in each bar show the number of patients in each group that had sore throat.

In this study we asked the patients in the three groups for sore throat 1 hour, 6 hours and 24 hours after the surgery. The incidence of sore throat was significantly lower in the betamethasone gel group compared with the IV dexamethasone and control groups at 1 h (P=0.05), 6 h (P=0.006), and 24 h (P=0.008) postoperatively (Fig.1). None of the patients in the betamethasone gel group reported a sore throat 24 h postoperatively. 

Bucking after extubation was observed in 14(18.7%), 8(10.7%), and 9(12%) patients in the IV dexamethasone, betamethasone gel, and control group respectively. 

The incidence of laryngospasm in the three groups was also determined. We had one case on the IV dexamethasone group, no cases in the betamethasone gel group and four cases in the control group. Statistical analysis showed no difference between the groups.

## Discussion

The major finding of the present study is that applying betamethasone gel is effective in reducing the incidence and severity of postoperative sore throat. The incidence of postoperative sore throat was significantly reduced when betamethasone gel was applied over the tracheal tube compared with the IV dexamethasone or standard saline group. Previous studies showed that the incidence of postoperative sore throat is approximately 6.6% and many factors may influence this incidence (2,3). 

Recently Epstein demonstrated that prophylactic corticosteroids decrease the risk of upper airway obstruction and probably the need for re-intubation (21). 

Corticosteroids reduce the synthesis of inflammatory mediators by inhibiting cyclo-oxygenase-2 during inflammation (22).

They also produce prostaglandins and leukotrienes that inhibit the phospholipase A2 through production of calcium-dependent phospholipid-binding proteins called annexins (23). Therefore the role of IV dexamethasone in reducing sore throat and cough has been previously proved by different studies (15-17). Previous studies by Ayoub et al and Selvaraj et al compared betamethasone gel against a control (4,18). They reported the beneficial effects of the steroid gel when applied to all portions of the tube that come into contact with the posterior pharyngeal wall, vocal cords, and trachea, and not just to the tip and cuff of the tracheal tube. 

Sumathi et al evaluated the application of betamethasone gel on the tracheal tube and compared it to lidocaine jelly in reducing postoperative sore throat (20). These patients underwent elective surgeries under general anesthesia and had no record of bucking on the tracheal tube at the time of extubation. The authors concluded that widespread application of betamethasone gel on the tracheal tube decreases the incidence and severity of postoperative sore throat. 

Our findings confirmed the results of previous studies, proving that the widespread application of betamethasone gel significantly reduces the incidence of postoperative sore throat. We opted for a different study design by selecting a larger sample size and added a control group in addition to the IV dexamethasone group. We also considered the extubation protocol in the different groups and recorded the incidence of bucking on the tracheal tube at the time of extubation. Our results showed a lower incidence of bucking at the extubation time for betamethasone gel groupcompared with the control and IV dexamethasone groups. 

Found a significantly lower incidence of sore throat in the betamethasone group compared with the IV dexamethasone group in the first, 6^th^, and 24^th^ hours postoperatively. We also showed that betamethasone gel was more effective in preventing sore throat incidence and severity in the 24^th^ hours postoperatively. It can be concluded that betamethasone gel had preventive effects on sore throat and needed a 24our period to manifest itself. This result was previously confirmed by Kazemi and Amini (10), as they considered betametha-sone gel in reducing sore throat, cough, and hoarseness after laryngo-tracheal intubation, However, this study had a smaller sample size (50 patients) and compared betametha- sone gel with placebo gel, 1 and 24 hours after extubation only.

## Conclusions

We conclude that betamethasone gel applied over the tracheal tube effectively mitigates postoperative sore throat compared with IV dexamethasone application.

## References

[1] Bilotta F, Branca G, Lam A, Cuzzone V, Doronzio A, Rosa G (2008). Endotracheal lidocaine in preventing endotracheal suctioning-induced changes in cerebral hemodynamics in patients with severe head trauma. Neurocrit Care.

[2] Kloub R (2001). Sore throat following tracheal intubation. Middle East J Anesthesiol.

[3] Ruangsin S, Wanasuwannakul T, Pattaravit N, Asim W (2012). Effectiveness of a preoperative single dose intravenous dexamethasone in reducing the prevalence of postoperative sore throat after endotracheal intubation. J Med Assoc Thai.

[4] Sumathi PA, Shenoy T, Ambareesha M, Krishna HM (2008). Controlled comparison between betamethasone gel and lidocaine jelly applied over tracheal tube to reduce postoperative sore throat, cough, and hoarseness of voice. Br J Anaesth.

[5] Stone DJ, Gal TJ, Miller RD (ed) (2000). Airway management. Anesthesia.

[6] Henderson J, Miller RD (2010). Airway management in the adult: Vol. II. Anesthesia.

[7] Ahmed A, Abbasi S, Ghafoor HB, Ishaq M (2007). Postoperative sore throat after elective surgical procedures. J Ayub Med Coll Abbottabad.

[8] Roscoe A, Kanellakos GW, McRae K, Slinger P (2007). Pressures exerted by endobronchial devices. Anesth Analg.

[9] Kazemi A, Amini A (2007). The effect of betamethasone gel in reducing sore throat, cough, and hoarseness after laryngo-tracheal intubation. Middle East J Anesthesiol.

[10] Navarro LH, Braz JR, Nakamura G, Lima RM, Silva Fde P, Módolo NS (2007). Effectiveness and safety of endotracheal tube cuffs filled with air versus filled with alkalinized lidocaine: a randomized clinical trial. Sao Paulo Med J.

[11] McHardy FE, Chung F (1999). Postoperative sore throat: cause, prevention and treatment. Anaesthesia.

[12] Tanaka Y, Nakayama T, Nishimori M, Sato Y, Furuya H (2009). Lidocaine for preventing postoperative sore throat. Cochrane Database Syst Rev.

[13] Ayoub CM, Ghobashy A, Koch ME, McGrimley L, Pascale VP, Qadir S (1998). Widespread application of topical steroids to decrease sore throat, hoarseness, and cough after tracheal intubation. Anesth Analg.

[14] Honarmand A, Safavi M (2008). Beclomethasone inhaler versus intravenous lidocaine in the prevention of postoperative airway and throat complaints: a randomized, controlled trial. Ann Saudi Med.

[15] Elhakim M, Ali NM, Rashed I, Riad MK, Refat M (2003). Dexamethasone reduces postoperative vomiting and pain after pediatric tonsillectomy. Can J Anaesth.

[16] Thomas S, Beevi S (2007). Dexamethasone reduces the severity of postoperative sore throat. Can J Anaesth.

[17] Park SH, Han SH, Do SH, Kim JW, Rhee KY, Kim JH (2008). Prophylactic dexamethasone decreases the incidence of sore throat and hoarseness after tracheal extubation with a double-lumen endobronchial tube. Anesth Analg.

[18] Selvaraj T, Dhanpal R (2002). Evaluation of the application of topical steroids on the endotracheal tube in decreasing postoperative sore throat. J Anaesthesiol Clin Pharmacol.

[19] McHardy FE, Chung F (1999). Postoperative sore throat: cause, prevention and treatment. Anaesthesia.

[20] Sumathi PA, Shenoy T, Ambareesha M, Krishna HM ( 2008). Controlled comparison between betamethasone gel and lidocaine jelly applied over tracheal tube to reduce postoperative sore throat, cough, and hoarseness of voice. Br J Anaesth.

[21] Epstein SK (2007). Corticosteriods to prevent postextubation upper airway obstruction: the evidence mounts. Crit Care.

[22] Lubenow TR, Ivankovich AD, Mc Carthy RJ, Barash PG, Cullen BF, Stoelting RK (2001). Management of acute postoperative pain. Clinical Anesthesia.

[23] Yao XL, Cowan MJ, Gladwin MT, Lawrence MM, Angus CW, Shelhamer JH (1999). Dexamethasone alters arachidonate release from human epithelial cells by induction of p11 protein synthesis and inhibiting phospholipase A2 activity. J Biol Chem.

